# The Use of Solutions of Corrossive Sublimate

**Published:** 1891-05

**Authors:** 


THE USE OF SOLUTIONS OF CORROSSIVE SUBLIMATE.—Dr. Ahb has published some observations to the effect that the antiseptic and germicidal effects of the above solution are markedly enhanced by employing them at a temperature of a 100 degrees F., and upwards. For instance, the effect of a one in ten or twenty thousand of the salt at this temperature is superior as a lactericide to a cold solution of one in five hundred. This is a fact of some importance, for the weaker the solution that can be employed with the assurance of obtaining the desired antiseptic effects, the better for the patient, seeing that the caustic action of the stronger preparations is undesirable, because injurious to the tissues. This is particularly the case in operations on serious cavities, such as the pleura and peritoneum.—*Medical Press.*

